# *cis*-Diamminedichloridoplatinum(II) *N*,*N*-dimethyl­formamide monosolvate

**DOI:** 10.1107/S1600536812024014

**Published:** 2012-06-02

**Authors:** Dean H. Johnston, Nathanael A. Miller, Cory B. Tackett

**Affiliations:** a Department of Chemistry, Otterbein University, Westerville, OH 43081, USA

## Abstract

In the title compound, *cis*-[PtCl_2_(NH_3_)_2_]·C_3_H_7_NO, the metal complex mol­ecules are stacked parallel to the *b* axis, forming close Pt⋯Pt inter­actions of 3.4071 (7) and 3.5534 (8) Å and weak N—H⋯Cl hydrogen bonds between the ammine ligand and the Cl atoms of the neighboring complex. Conventional N—H⋯O hydrogen bonds are formed between ammine ligands and the O atom of adjacent *N*,*N*-dimethyl­formamide mol­ecules. The crystal was found to be a split crystal and was analyzed using two domains related by a rotation of *ca* 4.4° about the reciprocal axis (−0.351 1.000 0.742) and refined to give a minor component fraction of 0.084 (6).

## Related literature

For a review of platinum anti­cancer coordination compounds, see: Reedijk (2009 ▶). For the preparation of *cis*-diamminedichloridoplatinum(II), see: Kukushikin *et al.* (1998 ▶). For single-crystal X-ray and neutron diffraction studies of *cis*-diamminedichloridoplatinum(II), see: Milburn & Truter (1966 ▶); Ting *et al.* (2010 ▶). For vibrational studies, see: Nakamoto *et al.* (1965 ▶). For crystallographic studies of dimethyl­formamide solvates and complexes of *cis*-diamminedichlorido­platinum(II) and related compounds, see: Raudaschl *et al.* (1983 ▶, 1985 ▶); Raudaschl-Sieber *et al.* (1986 ▶); Alston *et al.* (1985 ▶). For a crystallographic study of palladium analogs, see: Kirik *et al.* (1996 ▶). For a detailed analysis of linear chainstructures in platinum(II) complexes, see: Connick *et al.* (1997 ▶). For an analysis of hydrogen bonding in platinum–ammine complexes, see: Brammer *et al.* (1987 ▶).
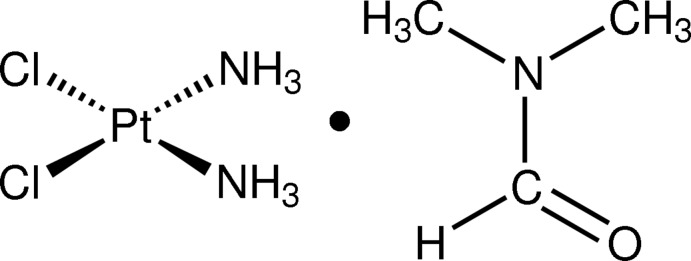


## Experimental

### 

#### Crystal data

[PtCl_2_(NH_3_)_2_]·C_3_H_7_NO
*M* *_r_* = 373.15
Triclinic, 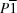

*a* = 6.2344 (9) Å
*b* = 6.8196 (11) Å
*c* = 11.5833 (18) Å
α = 105.285 (4)°
β = 96.061 (4)°
γ = 97.809 (4)°
*V* = 465.47 (12) Å^3^
*Z* = 2
Mo *K*α radiation
μ = 15.59 mm^−1^
*T* = 200 K
0.46 × 0.36 × 0.10 mm

#### Data collection

Bruker SMART X2S benchtop diffractometer
Absorption correction: multi-scan (*TWINABS*; Bruker, 2009 ▶) *T*_min_ = 0.05, *T*_max_ = 0.30
7440 measured reflections
1620 independent reflections
1484 reflections with *I* > 2σ(*I*)
*R*_int_ = 0.043

#### Refinement

*R*[*F* ^2^ > 2σ(*F* ^2^)] = 0.028
*wR*(*F* ^2^) = 0.072
*S* = 1.07
1620 reflections
96 parameters
H-atom parameters constrained
Δρ_max_ = 1.44 e Å^−3^
Δρ_min_ = −2.08 e Å^−3^

### 

Data collection: *APEX2* and *GIS* (Bruker, 2009 ▶); cell refinement: *SAINT* (Bruker, 2009 ▶); data reduction: *SAINT*; program(s) used to solve structure: *SHELXS97* (Sheldrick, 2008 ▶); program(s) used to refine structure: *SHELXL97* (Sheldrick, 2008 ▶) and *OLEX2* (Dolomanov *et al.*, 2009 ▶); molecular graphics: *PLATON* (Spek, 2009 ▶), *Mercury* (Macrae *et al.*, 2008 ▶) and *POV-RAY* (Cason, 2004 ▶); software used to prepare material for publication: *publCIF* (Westrip, 2010 ▶).

## Supplementary Material

Crystal structure: contains datablock(s) I, global. DOI: 10.1107/S1600536812024014/pk2417sup1.cif

Structure factors: contains datablock(s) I. DOI: 10.1107/S1600536812024014/pk2417Isup2.hkl

Supplementary material file. DOI: 10.1107/S1600536812024014/pk2417Isup3.mol

Additional supplementary materials:  crystallographic information; 3D view; checkCIF report

## Figures and Tables

**Table 1:** Hydrogen-bond geometry (Å, °)

*D*—H⋯*A*	*D*—H	H⋯*A*	*D*⋯*A*	*D*—H⋯*A*
N1—H1*E*⋯Cl1^i^	0.91	2.51	3.389 (7)	161
N2—H2*E*⋯Cl2^i^	0.91	2.53	3.403 (7)	162
N1—H1*D*⋯O1^ii^	0.91	2.13	3.023 (9)	167
N2—H2*F*⋯O1^iii^	0.91	2.31	3.198 (9)	165

Symmetry codes: (i) 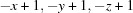; (ii) 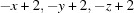; (iii) 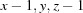.

**Fig. 1.**
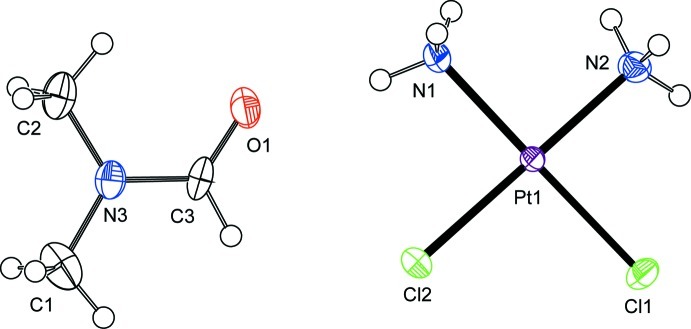
The molecular structure of the title compound drawn with 50% probability displacement ellipsoids for non-H atoms.

**Fig. 2.**
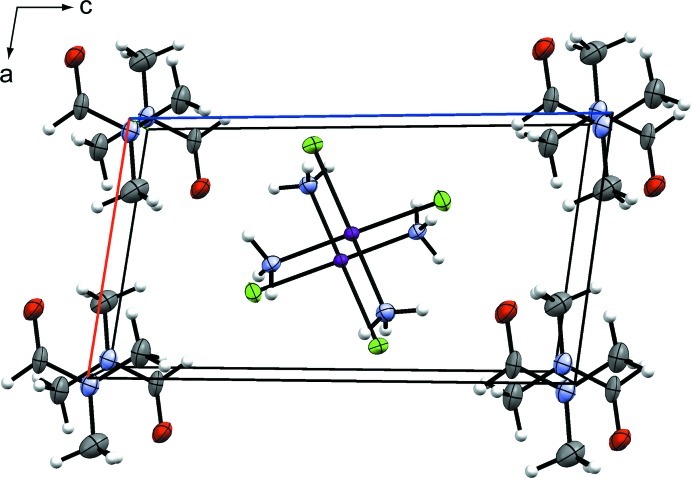
The packing of the title compound viewed along the b axis drawn with 50% probability displacement ellipsoids for non-H atoms.

**Fig. 3.**
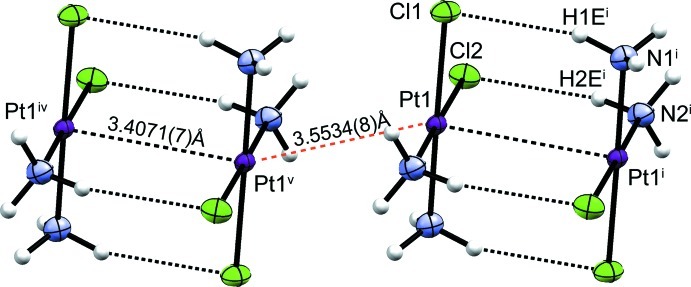
A view showing the platinum-platinum interactions and hydrogen bonding between the ammine ligands and the chlorine atoms on neighboring complexes. Additional donor/acceptor distances and angles are listed in Table 1. [Symmetry codes: (i) -x + 1, -y + 1, -z + 1; (iv) x, y + 1, z; (v) -x + 1, -y + 2, -z + 1.]

**Fig. 4.**
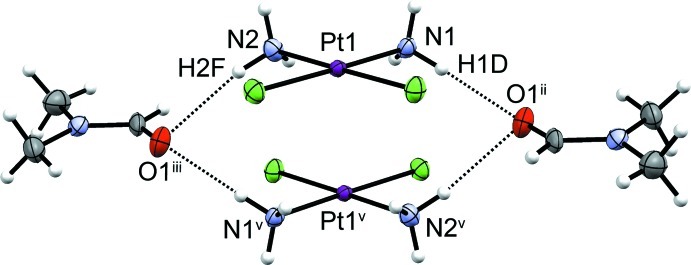
An illustration of the hydrogen bond interactions between the ammine ligands and the N,N-dimethylformamide solvent molecules. Additional donor/acceptor distances and angles are listed in Table 1. [Symmetry codes: (ii) -x + 2, -y + 2, -z + 2; (iii) x - 1, y, z - 1; (v) -x + 1, -y + 2, -z + 1.]

## supplementary crystallographic information

### Comment

*Cis*-diamminedichloridoplatinum(II), also known as cisplatin, is a widely used anti-cancer drug that has been extensively studied since its clinical applications were first discovered (Reedijk, 2009). The structure of cisplatin was first determined crystallographically in 1966 (Milburn & Truter, 1966); a recent study has elaborated the structure of the two common polymorphs (Ting *et al.*, 2010). A different DMF-solvate of cisplatin has been described previously (Raudaschl *et al.*, 1983, Raudaschl *et al.* 1985, Raudaschl-Sieber *et al.* 1986). A crown ether complex of cisplatin has also been crystallographically characterized (Alston *et al.*, 1985).

The title compound has a square-planar geometry (Fig. 1) and forms stacked chains along the crystallographic *b* axis with Pt—Pt distances of 3.4071 (7) and 3.5534 (8) Å and Pt—Pt—Pt angle of 156.90 (2) degrees as shown in Figures 2 and 3. Formation of extended platinum chains through stacking is a common motif in square-planar platinum compounds (Connick *et al.*, 1997). Hydrogen bonds are formed between the ammine hydrogen atoms and the chlorine atoms on the closer of the two neighboring complexes. This type of hydrogen bonding has been proposed or observed in previous studies on cisplatin and related complexes (Milburn & Truter, 1966; Brammer *et al.*, 1987; Ting *et al.*, 2010).

Additional hydrogen bonds are formed between the ammine hydrogen atoms and the oxygen atom of the *N*,*N*-dimethylformamide as illustrated in Figure 4. Similar interactions are seen in the DMF-solvated cisplatin structure previously described (Raudaschl *et al.*, 1983, Raudaschl *et al.*, 1985, Raudaschl-Sieber *et al.*, 1986). Interestingly, the previously described DMF solvate of cisplatin does not display stacking of the metal complexes seen in the title compound.

### Experimental

The title complex was prepared by refluxing 2.00 g (4.82 mmol) of K_2_[PtCl_4_] with 1.60 g (20.8 mmol) of ammonium acetate and 2.0 g (26.8 mol) of KCl in 25 ml of water for two hours, during which the solution changed from a dark red to green. The solution was hot filtered and a greenish-yellow powder formed on cooling. The powder was recrystallized by vapor diffusion of diethylether into a DMF solution of the complex yielding yellow-orange crystals. Infrared spectra were in agreement with literature values (Nakamoto *et al.*, 1965)

Data sets on multiple crystals showed evidence of at least two independent domains, either from agglomeration or from the crystal being cracked or split, perhaps during rapid cooling to 200 K.

### Refinement

The matrix relating the two domains, [1.007 0.033 - 0.041 - 0.010 1.013 - 0.022 0.118 0.060 0.975], corresponding to a rotation of 4.4 degrees about the reciprocal axis (-0.351 1.000 0.742), was determined using the *CELL_NOW* program (Bruker AXS, 2009). Integration and absorption correction (TWINABS, Bruker AXS 2009) gave 1558 unique reflections in domain 1, 1560 unique reflections in domain 2, and 211 unique overlapping reflections, or 14 percent overlapping reflections. The structure was solved using merged reflections from both domains (type HKLF 4 in SHELXL-97). The structure was refined using corrected reflections from only the major component including overlaps (type HKLF 5 in SHELXL-97).

Refinement produced a minor component fraction of 0.084 (6). All hydrogen atoms were positioned geometrically and refined with the atom positions constrained to appropriate positions with N—H distances of 0.91 Å and C—H distances of either 0.95 Å (amide) or 0.98 Å (methyl groups). A riding model was used for all H atoms with *U*_iso_(H) = 1.2 times *U*_iso_ (amine, amide) or 1.5 times *U*_iso_ (methyl carbon atoms).

### Crystal data

**Table:**

[PtCl_2_(NH_3_)_2_]·C_3_H_7_NO	*Z* = 2
*M**_r_* = 373.15	*F*(000) = 344
Triclinic, *P*1	*D*_x_ = 2.662 Mg m^−^^3^
*a* = 6.2344 (9) Å	Mo *K*α radiation, λ = 0.71073 Å
*b* = 6.8196 (11) Å	Cell parameters from 1090 reflections
*c* = 11.5833 (18) Å	θ = 3.3–24.8°
α = 105.285 (4)°	µ = 15.59 mm^−^^1^
β = 96.061 (4)°	*T* = 200 K
γ = 97.809 (4)°	Plate, clear yellow
*V* = 465.47 (12) Å^3^	0.46 × 0.36 × 0.10 mm

### Data collection

**Table:**

Bruker SMART X2S benchtop diffractometer	1620 independent reflections
Radiation source: fine-focus sealed tube	1484 reflections with *I* > 2σ(*I*)
Doubly curved silicon crystal monochromator	*R*_int_ = 0.043
Detector resolution: 8.3330 pixels mm^-1^	θ_max_ = 25.1°, θ_min_ = 3.1°
ω scans	*h* = −7→7
Absorption correction: multi-scan (TWINABS; Bruker, 2009)	*k* = −8→7
*T*_min_ = 0.05, *T*_max_ = 0.30	*l* = 0→13
7440 measured reflections	

### Refinement

**Table:**

Refinement on *F*^2^	Primary atom site location: structure-invariant direct methods
Least-squares matrix: full	Secondary atom site location: difference Fourier map
*R*[*F*^2^ > 2σ(*F*^2^)] = 0.028	Hydrogen site location: inferred from neighbouring sites
*wR*(*F*^2^) = 0.072	H-atom parameters constrained
*S* = 1.07	*w* = 1/[σ^2^(*F*_o_^2^)]
1620 reflections	(Δ/σ)_max_ = 0.001
96 parameters	Δρ_max_ = 1.44 e Å^−^^3^
0 restraints	Δρ_min_ = −2.08 e Å^−^^3^

### Special details

**Table:**

Refinement. Refinement of *F*^2^ against ALL reflections. The weighted *R*-factor *wR* and goodness of fit *S* are based on *F*^2^, conventional *R*-factors *R* are based on *F*, with *F* set to zero for negative *F*^2^. The threshold expression of *F*^2^ > σ(*F*^2^) is used only for calculating *R*-factors(gt) *etc*. and is not relevant to the choice of reflections for refinement. *R*-factors based on *F*^2^ are statistically about twice as large as those based on *F*, and *R*- factors based on ALL data will be even larger.

### Fractional atomic coordinates and isotropic or equivalent isotropic displacement parameters (Å^2^)

**Table:**

	*x*	*y*	*z*	*U*_iso_*/*U*_eq_	
Pt1	0.55286 (4)	0.74780 (4)	0.49265 (2)	0.01388 (13)	
Cl1	0.6813 (3)	0.6374 (3)	0.31075 (18)	0.0250 (4)	
Cl2	0.8971 (3)	0.7991 (3)	0.60324 (18)	0.0244 (4)	
N1	0.4361 (11)	0.8403 (11)	0.6522 (6)	0.0211 (15)	
H1D	0.5443	0.9266	0.7089	0.025*	
H1E	0.3894	0.7281	0.6772	0.025*	
H1F	0.3222	0.9076	0.6419	0.025*	
N2	0.2485 (11)	0.6999 (11)	0.3960 (6)	0.0236 (15)	
H2D	0.1706	0.7943	0.4345	0.028*	
H2E	0.1786	0.5706	0.3897	0.028*	
H2F	0.2604	0.7135	0.3208	0.028*	
O1	1.2567 (10)	0.8302 (10)	1.1506 (5)	0.0335 (15)	
N3	0.9601 (11)	0.7534 (9)	1.0048 (6)	0.0254 (17)	
C1	0.7242 (15)	0.7227 (15)	0.9760 (9)	0.041 (2)	
H1A	0.6598	0.7511	1.0510	0.061*	
H1B	0.6694	0.5797	0.9279	0.061*	
H1C	0.6836	0.8165	0.9294	0.061*	
C2	1.0852 (17)	0.7125 (15)	0.9063 (8)	0.037 (2)	
H2A	1.2411	0.7612	0.9374	0.055*	
H2B	1.0389	0.7845	0.8476	0.055*	
H2C	1.0608	0.5637	0.8667	0.055*	
C3	1.0583 (16)	0.8090 (13)	1.1198 (7)	0.027 (2)	
H3	0.9686	0.8339	1.1818	0.032*	

### Atomic displacement parameters (Å^2^)

**Table:**

	*U*^11^	*U*^22^	*U*^33^	*U*^12^	*U*^13^	*U*^23^
Pt1	0.01315 (19)	0.01376 (19)	0.01392 (18)	0.00092 (13)	0.00190 (12)	0.00322 (13)
Cl1	0.0266 (11)	0.0290 (11)	0.0192 (10)	0.0044 (9)	0.0089 (8)	0.0044 (8)
Cl2	0.0164 (10)	0.0311 (12)	0.0230 (10)	0.0032 (9)	−0.0005 (8)	0.0049 (9)
N1	0.018 (4)	0.022 (4)	0.019 (3)	−0.001 (3)	0.000 (3)	0.003 (3)
N2	0.020 (4)	0.029 (4)	0.023 (4)	0.005 (3)	0.003 (3)	0.010 (3)
O1	0.030 (4)	0.037 (4)	0.025 (3)	0.005 (3)	−0.005 (3)	−0.001 (3)
N3	0.031 (5)	0.020 (4)	0.022 (4)	0.003 (3)	0.001 (3)	0.003 (3)
C1	0.036 (6)	0.040 (6)	0.045 (6)	−0.001 (5)	−0.007 (5)	0.017 (5)
C2	0.049 (6)	0.038 (6)	0.022 (5)	0.008 (5)	0.001 (4)	0.009 (4)
C3	0.042 (6)	0.023 (5)	0.013 (4)	0.006 (4)	0.001 (4)	0.001 (4)

### Geometric parameters (Å, º)

**Table:**

Pt1—N1	2.034 (6)	N3—C3	1.339 (10)
Pt1—N2	2.037 (6)	N3—C2	1.437 (11)
Pt1—Cl1	2.3088 (19)	N3—C1	1.447 (11)
Pt1—Cl2	2.313 (2)	C1—H1A	0.9800
N1—H1D	0.9100	C1—H1B	0.9800
N1—H1E	0.9100	C1—H1C	0.9800
N1—H1F	0.9100	C2—H2A	0.9800
N2—H2D	0.9100	C2—H2B	0.9800
N2—H2E	0.9100	C2—H2C	0.9800
N2—H2F	0.9100	C3—H3	0.9500
O1—C3	1.229 (11)		
			
N1—Pt1—N2	91.8 (3)	C3—N3—C2	120.9 (8)
N1—Pt1—Cl1	178.99 (19)	C3—N3—C1	120.9 (8)
N2—Pt1—Cl1	87.6 (2)	C2—N3—C1	118.1 (8)
N1—Pt1—Cl2	87.9 (2)	N3—C1—H1A	109.5
N2—Pt1—Cl2	179.34 (18)	N3—C1—H1B	109.5
Cl1—Pt1—Cl2	92.63 (7)	H1A—C1—H1B	109.5
Pt1—N1—H1D	109.5	N3—C1—H1C	109.5
Pt1—N1—H1E	109.5	H1A—C1—H1C	109.5
H1D—N1—H1E	109.5	H1B—C1—H1C	109.5
Pt1—N1—H1F	109.5	N3—C2—H2A	109.5
H1D—N1—H1F	109.5	N3—C2—H2B	109.5
H1E—N1—H1F	109.5	H2A—C2—H2B	109.5
Pt1—N2—H2D	109.5	N3—C2—H2C	109.5
Pt1—N2—H2E	109.5	H2A—C2—H2C	109.5
H2D—N2—H2E	109.5	H2B—C2—H2C	109.5
Pt1—N2—H2F	109.5	O1—C3—N3	124.3 (8)
H2D—N2—H2F	109.5	O1—C3—H3	117.8
H2E—N2—H2F	109.5	N3—C3—H3	117.8
			
C2—N3—C3—O1	0.3 (12)	C1—N3—C3—O1	177.2 (8)

### Hydrogen-bond geometry (Å, º)

**Table:**

*D*—H···*A*	*D*—H	H···*A*	*D*···*A*	*D*—H···*A*
N1—H1*E*···Cl1^i^	0.91	2.51	3.389 (7)	161
N2—H2*E*···Cl2^i^	0.91	2.53	3.403 (7)	162
N1—H1*D*···O1^ii^	0.91	2.13	3.023 (9)	167
N2—H2*F*···O1^iii^	0.91	2.31	3.198 (9)	165

Symmetry codes: (i) −*x*+1, −*y*+1, −*z*+1; (ii) −*x*+2, −*y*+2, −*z*+2; (iii) *x*−1, *y*, *z*−1.
